# The effect of incorporating herbal (*Lippia javanica*) infusion on the phenolic, physicochemical, and sensorial properties of fruit wine

**DOI:** 10.1002/fsn3.2432

**Published:** 2021-06-25

**Authors:** Armistice Chawafambira

**Affiliations:** ^1^ Department of Food Science and Technology Chinhoyi University of Technology Chinhoyi Zimbabwe

**Keywords:** fruit wine, *Lippia javanica*, phytochemical, *Saccharomyces cerevisiae*, sensory quality

## Abstract

The use of medicinal herbs in food processing to improve food quality and human health is growing in sub‐Saharan Africa. Herbal infusions have perceived medicinal benefits. This study investigated the effect of incorporating *L. javanica* extract on the phenolic, physicochemical, and sensory properties of a *Uapaca* *kirkiana* fruit‐based wine. The fruit and *L. javanica* were analyzed for proximate, pH, total soluble sugars (TSS), total sugar, titratable acidity, phenolics, and antioxidant activity (AOA). The prepared substrate was fermented at optimized fermentation temperature of 23°C, pH of 3.4, inoculum concentration of 9.5% (v/v) (*Saccharomyces cerevisiae*), and *L. javanica* extract concentration of 10% (v/v). The produced wine had a pH, total sugar, TSS, total acidity, and vitamin C content of 3.1 ± 0.2, 3.0 ± 0.1 g/L, 4.8 ± 0.1%, 5.9 ± 0.01 g/L, and 4.4 ± 0.1 mg/100 g, respectively. The wine had 12.2 ± 2.1 mg GAE/g, 0.06 ± 0.01 mg/g, and 1.8 ± 1.1 mg CE/g total phenols, tannins, and flavonoids, respectively. The alcohol, free sulfur dioxide (SO_2_), AOA, and color values of the produced wine were 10.2 ± 0.1 alcohol by volume (ABV)%, 58.1 ± 1.2 mg/L, 73.1 ± 0.1 EC_50_ mg/L, and 40.4 ± 2.1% yellow, respectively. The flavor, color, and overall acceptance of the produced wine were rated as “good” and were significantly different (*p* < .05) from control wine. The addition of *L. javanica* extracts enhanced total phenol, color, and sensory properties of the wine. The utilization of *U. kirkiana* fruit by incorporating *L. javanica* infusion can reduce postharvest losses and improve nutrition and health.

## Graphical abstract

This research paper will provide an understanding of the use of herbal infusions and their functional importance in fruit wines. The study results indicated that herbal extracts from *L. javanica* enhanced the phytochemicals, antioxidant activity, color, and sensory properties of the wine. This leads to the promotion of the utilization of medicinal herbs as food additives in food processing with the aim of improving food quality and human health.

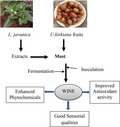

## 1 INTRODUCTION

**FIGURE 1:**
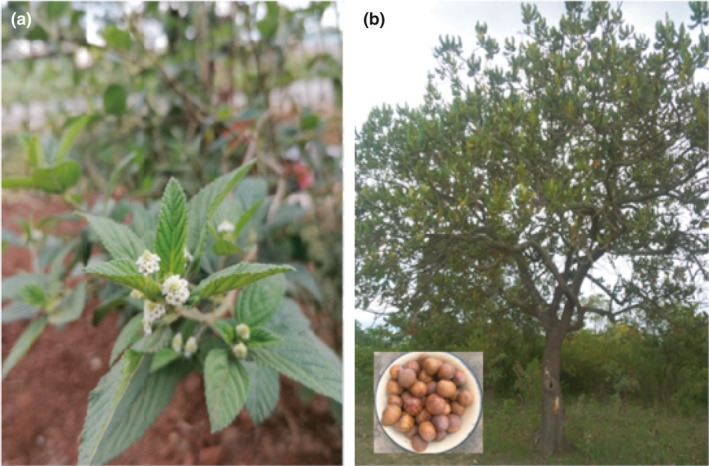
(a) *Lippia javanica* flowers and leaves. (b) *Uapaca kirkiana* fruit tree and fruits

In recent years, there has been an increase of interest in herbal infusions due to the effective promotion of their medicinal properties (Bhebhe et al., 2015). An underutilized indigenous herbal plant, *Lippia javanica,* has been made popular in many communal homes of Zimbabwe as a tisane (Maroyi, 2017). *Lippia javanica* (Burm.f.) Spreng belongs to the family Verbenaceae (Figure 1a) and is the most commonly occurring Lippia species in eastern central and southern Africa. The species is locally known as Zumbani (Shona)/umsuzwane (Ndebele) in Zimbabwe and has a long history of traditional uses as indigenous herbal tea (Bhebhe et al., 2016), food additive, health drink (Van Wyk, 2008), and health‐promoting plant when consumed in the body. *L. javanica* is rich in volatile oil, mainly carvone, caryophyllene, ipsdienone, ipsenone, limonene, linalool, myrcenone, myrcene, ocimenone, piperitenone, p‐cymene, tagetenone, and sabinene (Chagonda & Chalchat, 2015; Viljoen et al., 2005).

Many groups of phytochemicals including volatile and nonvolatile secondary metabolites such as alkaloids, flavonoids, amino acids, triterpenes, iridoids, and minerals have been identified from *L. javanica* (Maroyi, 2017). Many authors have reported on the antidiabetic (Arika et al., 2015), antioxidant (Shikanga et al. 2010), antimicrobial (Viljoen et al., 2005), and anticancer (Fouché et al., 2008) activity of *L. javanica*. *L. javanica* is currently being used as a herbal plant with many ethnomedicinal applications such as in asthma, bronchitis, chest pains, colds, cough, diarrhea, and fever (Maroyi, 2017). *Lippia javanica* plant has no health effect associated with its consumption in humans. *Lippia* *javanica* is used as a food additive in Kenya (Kipkore et al., 2014) and leafy vegetable in India (Narzary et al., 2015). Conversely, the use of mature *L. javanica* plant as a food additive and in food blends is still limited in Zimbabwe.

Fruits provide energy, minerals, vitamins, and phytochemicals, and their consumption improves the physiological functions and overall health in humans (Kaur et al., 2019). *Uapaca kirkiana* belongs to the genus *Uapaca* (Figure 1b) of the family *Euphorbiaceae* (World Agroforesty Centre, 2021) and subfamily *Phyllanthaceae* (Ecocrop Database, 2021) and is an underutilized indigenous fruit tree (IFT) that is well adapted to the miombo ecological zone in sub‐Saharan Africa (Chawafambira et al., 2020). The fruit is known from different countries by different names as wild loquat in English, nkusu/msuku in Tanzania, Zambia, and Malawi, and mahobohobo (Ndebele language) or mazhanje (Shona language) in Zimbabwe (Chawafambira et al., 2020). The fruit tree is found distributed in semidry and wet areas of Zimbabwe and it produces fruits which ripen from October to February (Chawafambira et al., 2020). Besides, Stadlmayr et al. (2013) reported the proximate composition of the fruit pulp as ash (1.1 g/100 g), carbohydrates (28.7 g/100 g), calories (523 kcal/kJ), fat (0.4 g/100 g), fiber (2.3 g/100 g), proteins (0.5 g/100 g), vitamin C (16.8 mg/100 g), and water (72.6 g/100 g).

Fruits wines have recently increased public interest as health‐enhancing functional foods (Tsegay & Gebremedhin, 2019). Studies have revealed the possibilities of developing health‐enhancing functional fruit wines through the incorporation of medicinal plants with fruit juice (Fracassetti et al., 2019; Lee & Chen, 2016). Globally, fermentation of grape and apple fruits is commonly used to commercially produce fruit wines and the fermentation of *U*. *kirkiana* fruit juice with added *L. javanica* leaf extracts has the potential to produce functional fruit wines. The production of fruit wines from other fruits other than grapes is gaining customer interest because they are considered as functional foods (Velić et al., 2018).

In Zimbabwe, *U. kirkiana* fruits have not yet been incorporated into industrial wine production. Also, *L. javanica* has not been incorporated in fruit wines. Maroyi (2017) reported the potential of using *L. javanica* in new food product development. Therefore, this study aimed at investigating the effect incorporating *L. javanica* infusion on the phenolic, physicochemical, and sensorial properties of fruit wine.

## 2 MATERIALS AND METHODS

### 2.1 Sample collection

Ripe *U. kirkiana* fruits that had fallen on the ground were collected from Bikita communal area. Bikita is a dry area located 20. 5 S° 31.37° E in Agro farming region 4 in Zimbabwe. *U. kirkiana* fruit trees are found distributed in the forests and some are domesticated at homes. *L. javanica* was collected from plants that were growing in the forest and were sun‐dried.

### 2.2 Pulp extraction and preparation of *Uapaca kirkiana* must

Ripe *U. kirkiana* fruits were washed thoroughly using distilled water to remove soil particles and then disinfected with sterile cotton wool soaked in ethanol (70% v/v). The edible portion of the fruits was then obtained by cutting the fruit, removing seeds, mashing, and sieving. The crude pulp mixture was sieved through an 800‐μM sieve in the laboratory to obtain pulp samples. The fruit pulp was mixed with distilled water (1:1 w/v). A weight of 75 mg of sodium metabisulfite (Na_2_S_2_O_2_) was added into 1L of must (fruit juice) and stirred. Microbial contamination and fermentation were prevented by the addition of the sodium metabisulfite into the juice samples before the addition of the starter culture. The juices samples were preserved at 4°C under refrigeration conditions.

### 2.3 Preparation of *Lippia javanica* extracts (infusion)

The extraction process was conducted following the water extraction method. Distilled water was used in the extraction process because it was found a better extraction solvent for phenolic compounds (Bhebhe et al., 2015). Sun‐dried *L*. *javanica* leaves were sorted and washed (twice) to remove any dirt particles. The leaves were then boiled in a stainless pot with 1L distilled water at 65–70°C for 8 min. The leaves were then strained, and the liquid extract was cooled and analyzed.

### 2.4 Preparation of Starter culture

Commercial yeast Anchor Wine Yeast (N96 *Saccharomyces cerevisiae bayanus*) was purchased from a local supermarket. The yeast was prepared according to the manufacturer's specifications which were 20–30 g/hl. A weight of 0.3 g dry anchor wine yeast cell was hydrated with 50 ml of warm distilled water at 35°C and added into the yeast extract peptone dextrose (YEPD) media. The mixture was then diluted to 150 ml using sterilized distilled water. This mixture was then incubated for 24 hr at 28°C and shaked at 44 × *g* in an Orbital digital rotary shaker (KS501, Thermo Fisher Scientific). The incubation temperature was used because yeast grows well at room temperature range of 25–28°C but not at high temperature above 30°C. The mixture was then transferred into a 1‐L Erlenmeyer flask which was cleaned with cotton wool that was soaked in 1% sulfur dioxide and had 500 ml of the sterilized *U. kirkiana* juice pulp. The mixture was adjusted by the addition of 200 g/L sugar, 70 mg/L sodium thiosulfate, and lemon juice to a pH of 3.4. The mixture was then incubated for 36 hr at 28°C and ready for use in the fermentation process.

### 2.5 Fermentation of must to wine, clarification, and filtration

Fermentation, clarification, and filtration of the fruit wine samples were carried out according to the method described by Tsegay and Gebremedhin (2019) with slight modification. Primary fermentation was initiated in wine by the addition of the starter culture. One (a) liter capacity fermentation vessels (Erlenmeyer flasks) were prepared by soaking in a 1% sulfur dioxide (19 g/L sodium metabisulfite) solution for 1 hr and then rinsed with distilled water. A 1 L of prepared *U. kirkiana* juice was then poured into the fermentation vessels under aseptic conditions. The sugar content of the substrate was then adjusted by addition of table sugar (200 g), pH of 3.4 (using 0.5 g/L fresh lemon juice), and sulfur thiosulfate (70 mg/L). The adjusted pH of 3.4 was used in this study because it inhibits spoilage bacteria and wine yeast can metabolize at this pH level. *L*. *javanica* extract and inoculum (propagated yeast) were added at a concentration of 10% (v/v) and 9.5% v/v of 8.2 Log CFU/mL viable cells, respectively. This was done to ensure rapid onset of fermentation of the must which had high sugar content. The control experiments had no *L. javanica* extract added. The fermentation vessels were closed using cotton wool that was soaked in 1% sulfur dioxide. Secondary fermentation was done in an air‐tight flask at 23°C and monitored for 10 days. This temperature was used to promote a steady growth of yeast cells and controls the levels of volatile compounds. After fermentation, the wine was chilled to 5°C, racked with minimum exposure to the air, and clarified. The fermented wine was then filtered using a sterilized cotton cheesecloth. The wine was then packaged into 330‐mL sterilized glass bottles and then allowed to mature at 15–24°C.

### 2.6 Chemical analysis

Proximate tests on crude protein, moisture content, crude fat, crude fiber, and ash content were determined according to the Association of Official Analytical Chemists (AOAC) using Kjeldahl (AOAC method 991.20) (AOAC method 925.45), using Soxhlet method (AOAC 989.05), using the enzymatic gravimetric method (AOAC method 985.29), and using dry ashing (AOAC method 938.08), respectively. Mineral content and carbohydrate content were analyzed using inductively coupled plasma optical emission spectrometer (ICP‐OES) (Agilent 5100, Agilent Technologies) and difference method, respectively (AOAC, 2005). The vitamin C (ascorbic acid) content was determined by the dichlorophenolindophenol (DCPIP) titration method (AOAC, 2005). The total sugar content was determined using the sulfuric acid method described by Debebe et al. (2018).

### 2.7 Physicochemical properties of the Fruit Juice and Wine

#### 2.7.1 pH and TSS

The pH and TSS of the fruit juice and the prepared wine were determined according to AOAC (2005) standard methods using a digital pH meter (BT‐675, BOECO, Hamburg, Germany) and digital refractometer (MA871, North Carolina, Milwaukee Instruments, USA). During the pH and TSS measurement, 10 ml of fruit juice and wine samples were analyzed.

#### 2.7.2 Total acidity

The Total acidity (TA) was determined according to a standard method by AOAC (2005). The samples were dissolved in 100 ml distilled water and titrated against 0.1 M NaOH solution. The production of pink color was recorded as the endpoint using phenolphthalein as an indicator.

#### 2.7.3 Extraction of phenolic compounds

Dried *U. kirkiana* fruit pulp and plant material of the *L. javanica* leaves were ground into a powder and sieved through a < 500 microns sieve into powder. Extraction of the soluble phenolic compounds was done using methanol: acetone: water (7:7:1 v/v/v), and the supernatant (extract) was stored at 4°C for analysis.

#### 2.7.4 Total phenol

Total phenolic content was determined using a method described by Harbone (1998) and Makkar (1999). A 50 µl sample of extracts, fruit pulp extract, and wine was diluted with 1L distilled water. Sodium carbonate (2.5 ml) and 1 N Folin‐C reagent (500 µl) and were then added, and the mixture was incubated at 25°C for 40 min. The absorbance of the mixture was recorded at 725 nm using a Spectronic Genesys spectrophotometer (Genesys 5, Thermo Fisher Scientific) against a methanol blank. A calibration equation (*y* = 0.0013*x* + 0.0177; *R* ^2^ = .9982) was produced using standard gallic acid (Merck) and was used to calculate the total phenolic content in micrograms of gallic acid equivalence (GAE) as (µg GAE/ml Dry weight (DW) of the sample.

#### 2.7.5 Tannin binding assay

Tannins were determined following the method adapted from Makkar (1999). One gram of polyvinylpolypyrrolidone (PVPL) was dissolved in 1 ml distilled water. A 1 ml sample was then added, and this mixture was vortexed and incubated for 15 min at 4°C. The mixture was then centrifuged at 1,107 *g* using a Microyn Digital Bench‐top Centrifuge. The total content of phenolic compounds in the supernatant was determined by measuring the absorbance at 725 nm using a Spectronic Genesys spectrophotometer (Genesys 5, Thermo Fisher Scientific). The tannin content was calculated as follows:

$$\text{Tannin}\left(\frac{\text{mg}}{g}\right)=A_{1}‐A_{2}$$

where A_1_ = total content of phenolic compounds before binding with PVPL and A_2_ = total content of phenolic compounds after binding with PVPL.

#### 2.7.6 Flavonoid content

Flavonoid content was determined using the vanillin assay (AOAC, 2005), where 0.5 ml of the sample was measured and distilled water was added to make 1 ml in a test tube. The mixture was mixed with 1:1 v/v methanol‐HCl (2.5 ml) and then 0.5 g/25 ml vanillin reagent (2.5 ml) was added. The mixture was then vortexed and left to stand for 20 min. The absorbance was done at 500 nm using a Spectronic Genesys spectrophotometer (Genesys 5, Thermo Fisher Scientific) and 50% methanol as a blank. The total flavonoid (proanthocyanidins) content was calculated and expressed as catechin equivalent (CE) per g dry weight.

#### 2.7.7 Antioxidant activity (DPPH free radical scavenging)

The radical scavenging activity was determined according to the AOAC method (AOAC, 2005). In the original experiment, absorbance was done at 515 nm. A 3.9 ml solution of 2,2‐diphenyl‐1‐picrylhydrazyl (DPPH) radical solution (0.06 mm) was mixed to 100 μl sample and incubated in the dark for 1 hr at 25°C. Ascorbic acid was used as a reference compound. The absorbance was determined at 517 nm using a Spectronic Genesys spectrophotometer (Genesys 5, Thermo Fisher Scientific) after calibration with methanol. The DPPH radical scavenging activity was expressed in terms of EC50 (effective concentration at which the DPPH radical was scavenged by 50%).

#### 2.7.8 Color of the wine

The color of the prepared fruit wine was measured using a UV–Vis spectrophotometer (Genesys 10S, Thermo Scientific) with 1 cm path length cuvettes at absorbances 420 nm, 520 nm, and 620 nm following a method described by Yildirim (2006). Color density (A_420nm_ + A_520nm_), color intensity (IC) (A_420nm_ + A_520nm_ + A_620nm_), and shade (A_420nm_/A_520nm_) were used to describe the color of the wine using distilled water as a blank. The levels of blue (% B) red (% R), and yellow (% Y) were determined as A_620nm_ × 100/IC, A_520nm_ × 100/IC, and A_420nm_ × 100/IC, respectively.

#### 2.7.9 Free sulfur dioxide

The free sulfur dioxide was determined following a method described by the International Organisation of Vine and Wine, formerly the Organisation Internationale de la Vigne et du Vin (O.I.V.) (2016). A wine sample (50 ml) was transferred into a 500‐mL conical flask containing 30mg ethylenediaminetetraacetic acid (EDTA), 5 ml starch solution (5 g/L), and 3 ml of 10% sulfuric acid. The mixture was shaked and the resulting solution was then titrated with 0.025 M iodine solution. The production of a blue color that lasted for 10 s was used as the endpoint. The volume of iodine used was recorded. Free sulfur dioxide was then calculated by multiplying the volume of iodine by 32 in milligrams per liter.

#### 2.7.10 Alcohol content

In determining the ABV, each wine sample was distilled according to the procedure by ‐Tsegay et al. (2018) with slight modification. In this experiment, 150 ml of the wine sample was used. After distillation, the collected distillate was cooled at room temperature and distilled water was then added until the volume reached 150 ml. The original gravity (before fermentation) of the must and the final gravity of the distilled wine (after fermentation) were determined using a specific gravity hydrometer (Fisherbrand, Thermo Fisher Scientific, Canada) at room temperature. The ABV was then calculated using the following equation:

$$\text{ABV}\left(\%\right)=\frac{1.05}{0.79}\left(\frac{\text{Original gravity}‐\text{Final gravity}}{\text{Final gravity}}\right)\times 100.$$

where 1.05 g is the amount of carbon dioxide produced for every gram of ethanol produced and 0.79 g/ml is the density of ethanol alcohol.

### 2.8 Sensory evaluation

In the sensory evaluation process, 30 semitrained panelists were selected and analyzed the sensory characteristics of the prepared wine samples. The panelists had different age‐groups: 20–29, 30–39, 40–49, and 50+ years. The number of females and males was 6 and 9, respectively. Panelists were randomly provided with 30 ml of wine samples filled into 150‐ml wine glasses. The samples were coded using a three‐letter code. Each panelist was instructed to evaluate for the taste, color, flavor, and overall acceptability of each sample in triplicate. A 5‐point hedonic scale with the following key: 1 = very bad, 2 = bad, 3 = average, 4 = good, and 5 = very good, was used to indicate panelist score. Panelists were placed in individual booths and were also instructed not to discuss their results during the sensory evaluation process.

### 2.9 Data analysis

All results were expressed as mean ± *SD*, and analysis was carried out using Statistical Package for the Social Sciences (SPSS) package version 18.0 (Coakes and Ong, John Wiley & Sons) at 5% level of significance. Physiochemical properties results were analyzed using one‐way analysis of variance (ANOVA). The Mann–Whitney U test was used to analyze the observed differences in the means of wine attributes.

## 3 RESULTS AND DISCUSSION

### 3.1 Chemical, phenolic, and mineral composition of *Lippia javanica* leave and *Uapaca kirkiana* fruit

Proximate composition on *L. javanica* showed higher ash, carbohydrate, fat, fiber, protein, and moisture content of 6.1 ± 1.3, 64.1 ± 0.4, 64.1 ± 0.4, 6.3 ± 0.1, 11.7 ± 0.2, and 11.2 ± 1.2%, respectively, when compared to *U*. *kirkiana* fruit pulp (Table 1). Furthermore, the *L*. *javanica* leaves are good sources of minerals such as calcium, potassium, phosphorus, copper, iron, magnesium, and zinc (Mahlangeni et al., 2018). The presence of these essential minerals in the leaves has made the plant important in human nutrition as herbal tea and food additive (Maroyi, 2017). More so, the significant concentrations of these minerals could enhance the nutritional and curative properties of *L. javanica*. *L. javanica* had a higher radical scavenging activity of 64.2 ± 0.1 mg/L than *U. kirkiana* (35.1 ± 0.1 mg/L) (Table 1). This could be explained by the presence of higher total phenolic content in comparison with *U. kirkiana* fruit which is a popular indigenous fruit tree that is consumed by many people in Zimbabwe (Chawafambira et al., 2020). Additionally, Shikanga et al. (2010) reported a higher phenolic content of 14.8 mg GAE/g DW in leaf extracts of *L. javanica*.

**TABLE 1:** Nutritional composition of *Lippia* *javanica* leaves and *Uapaca* *kirkiana* fruit pulp

Composition	*L. javanica* leaves	*U. kirkiana* fruit pulp
Moisture content (%)	11.2 ± 1.2^a^	69.0 ± 2.3^b^
Ash (%)	6.1 ± 1.3^b^	1.0 ± 0.1^a^
Crude fat (%)	64.1 ± 0.4^b^	0.4 ± 0.1^a^
Crude fiber (%)	6.3 ± 0.1^b^	0.9 ± 0.2^a^
Crude protein (%)	11.7 ± 0.2^a^	0.3 ± 0.1^b^
Carbohydrate (%)	64.1 ± 0.4^a^	28.4 ± 0.2^b^
Total phenols (mg GAE/g)	14.3 ± 0.2^a^	0.074 ± 0.002^b^
Tannins (mg/g)	0.02 ± 0.01^a^	0.01 ± 0.01^a^
Flavonoids (mg CE/g)	2.4 ± 0.2^b^	0.011 ± 0.01^a^
Antioxidant activity (EC_50_ mg/L)	64.2 ± 0.1^a^	35.1 ± 0.1^b^
Minerals (mg/100 g)		
Fe	77.3 ± 0.86^a^	12.2 ± 0.34^b^
Ca	485.2 ± 0.1^a^	17.1 ± 0.05^b^
Zn	3.8 ± 0.2^a^	0.90 ± 0.07^b^
Cu	1.1 ± 0.1^a^	0.86 ± 0.01^a^
K	1631 ± 0.05^a^	446 ± 121.2^b^
P	410 ± 0.50^b^	23.2 ± 0.21^a^
Mg	267 ± 0.35^a^	37.2 ± 0.05^b^

Note

Mean ± *SD* are reported. Means in a row with different superscripts (^a, b^) are significantly different at *p* < .05.

### 3.2 Physiochemical properties of fruit juice, *Lippia javanica* leaf extract, and wines

The pH, °Brix, and total acidity of fruit juice, herbal infusion, and the prepared wines are shown in Table 2. The fruit juice and *L. javanica* leave extract had pH of 4.4 ± 0.05 and 5.8 ± 0.1, respectively. These pH values indicated that further pH adjustment was required before they were used as substrates for wine fermentation. This resulted in fresh lemon being added in this experiment to adjust to a pH of 3.4. The low pH observed in the wine could also be attributed to the physiological action of yeasts, lactic acid bacteria, and organic acids that are produced during alcoholic fermentation (Tsegay & Gebremedhin, 2019). The pH differences between the wine with herbal infusions and control wine sample were significantly different at *p* < .05.

**TABLE 2:** Physiochemical properties of *Uapaca* *kirkiana* fruit juice, *Lippia* *javanica* leaf extract, and wines

Parameter	*U. kirkiana* fruit juice	*L. javanica* leaf extract	Wine with *L. javanica* leaf extracts	Control wine	*p*‐Value
pH	4.4 ± 0.05^b^	5.8 ± 0.1^c^	3.1 ± 0.2^a^	2.8 ± 0.1^a^	.021
Total soluble solids (%)	22.3 ± 0.12^b^	4.1 ± 1.01^a^	4.8 ± 0.1^a^	4.2 ± 0.01^a^	.010
Total sugar (g)	21.1 ± 1.02^c^	1.1 ± 0.03^a^	3.0 ± 0.1^b^	2.4 ± 0.2^a^	.01
Total acidity (g/L)	0.37 ± 0.07^a^	0.12 ± 0.10^a^	5.9 ± 0.01^c^	4.8 ± 0.01^b^	.036
Vitamin C (mg)	16.1 ± 0.5^b^	17.2 ± 0.2^b^	4.4 ± 0.1^a^	3.3 ± 0.1^a^	.013
Dry matter (g)	27.1 ± 0.1^b^	12.1 ± 1.2^a^	ND	ND	.01

Note

Mean ± *SD* are reported. Means in a row with different superscripts (^a, b, c^) are significantly different at *p* < .05.

The total soluble solids (TSS) of *L. javanica* extract and *U. kirkiana* fruit juice were 4.1 ± 1.01% °Brix and 22.3 ± 0.12% °Brix, respectively. Katsvanga et al. (2007) noted that fruits that grow in humid conditions with warm nights often have higher TSS levels and lower fruit acidity, which could explain the observed results on total soluble solids and TA on *U. kirkiana* fruit juice used in this study. The fruits were collected from a semiarid and hot area with maximum temperatures >29°C. Also, the TSS could be due to the effect of solar radiation during fruit ripening stage, which promotes the increase in the conversion of starch to sucrose, and reduction in sugars (Alston, 1992). Most fruits other than grapes have lower sugar content and table sugar (sucrose) had to be added to the must before fermentation. The fruits used in this experiment had a sugar content of 21.1%. The main sugar present in fruits is sucrose which is fermented by yeast producing major metabolites ethanol, glycerol, and carbon dioxide (Velić et al., 2018). The produced wine had sugar content of 3 g and could be explained as the residual sugars that remain unfermented in the wine (Velić et al., 2018). The prepared wine had a low total soluble solid content of 4.8 ± 0.1%. This was due to the solubilization of dissolved solids such as sugars as they were broken down via a series of biochemical reactions to produce alcohol, organic acids, and carbon dioxide (Tsegay & Gebremedhin, 2019).

The total acidity was lower in *L. javanica* extracts (0.12 ± 0.10) but increased to 0.31 ± 0.01 in the prepared wine. The high acid content in the *U. kirkiana* fruit could be attributed to the presence of organic acids (Chen et al., 2009). Vertuani et al. (2002) supported the explanation and reported the occurrence of tartaric, citric, malic, succinic, and ascorbic acid in ripe fruit pulps. The organic acid composition is determined by the fruit species, climate and geomorphological property of soil (Velić et al., 2018). As a result, the acidity of fruit used in winemaking affects the acidity of the fruit wine. The wine with *L. javanica* extract had a lower total titratable acidity of 5.9 g/L when compared to wines produced from orange fruit (6.3 g/L) and kiwi fruit wine (13.6 g/L) (Kelebek et al., 2009; Soufleros et al., 2001). This could be attributed to the differences in concentrations of organic acids especially citric acid which is present in most citrus fruits. Velić et al. (2018) reported the presence of malic acid as a major organic acid present in apple (6.2 g/L), blackberry (3.5 g/L), and cherry (6.8 g/L) wines.

The *L. javanica* leaves had higher antioxidant activity than the fruit as shown in Table 1. The free radical scavenging activity of *L. javanica* can be explained by the presence of phenolic compounds which contain reactive properties in their chemical structures for free radical scavenging activity (Chanda and Dave, 2009). Muchuweti et al. (2006) reported that ethanolic leaf extract of *L. javanica* had a 74.4% inhibition of the DPPH radical. Also, Bhebhe et al. (2016) found similar activity in *L. javanica* leaves.

### 3.3 Alcohol, color, sulfur dioxide, and phenolic content of wine

The total phenolic composition of the fruit, *L. javanica,* and the produced wines is shown in Table 3. The *L. javanica* had a high total phenol content and suggests the presence of phenolic compounds. Olivier et al. (2010) reported the presence of phenolic compounds and their derivatives such as 3,4‐dihydroxy‐β‐phenylethoxy‐O‐[6"‐β‐caffeoyl‐α rhamnopyranosyl‐(1"',3")‐O‐β‐glucopyranoside], commonly known as isoverbascoside 3, and coumarin 1, 3,4‐dihydroxy‐β‐phenylethoxy‐O‐[4"‐β‐caffeoyl‐α‐rhamnopyranosyl‐(1"',3")‐O‐β‐glucopyranoside], commonly known as verbascoside 2. Mujovo et al. (2008) observed the presence of flavanones in extracts of *L. javanica* leaves such as apigenin 7, 6‐methoxyluteolin 3', 4', 7‐trimethyl ether 10, 6 methoxyluteolin 4'‐methyl ether 9, and cirsimaritin 8.

**TABLE 3:** Alcohol, color, and sulfur dioxide (SO_2_) content of wine

Composition	Wine with *Lippia* *javanica* leaf extract	Control
Alcohol (ABV %)	10.2 ± 0.1^a^	8.6 ± 0.02^b^
Free SO_2_ (mg/L)	58.1 ± 1.2^a^	50.2 ± 2.1^a^
Antioxidant activity (EC_50_ mg/L)	72.1 ± 0.1^a^	46.3 ± 0.2^b^
Color		
Color density (A_420nm_ + A_520nm_)	0.80 ± 0.05^a^	0.72 ± 0.01^a^
Color intensity (A_420nm_ + A_520nm_ + A_620nm_)	0.93 ± 0.01^a^	0.88 ± 0.01^a^
Shade/Brightness (A_420nm_/A_520nm_)	1.51 ± 0.01^a^	1.12 ± 0.03^b^
% R	30.2 ± 1.3^a^	23.1 ± 0.2^b^
% Y	40.4 ± 2.1^a^	32.2 ± 0.3^b^
% B	1.39 ± 0.03^a^	1.13 ± 0.01^a^
Phenolic compounds		
Total phenols (mg GAE/g)	12.2 ± 0.2^a^	0.1 ± 0.1^b^
Tannin (mg/g)	0.06 ± 0.01^a^	0.02 ± 0.01^a^
Flavonoids (mg CE/g)	1.8 ± 0.1^a^	0.04 ± 0.01^b^

Note

B, R, and Y refer to proportions of blue, red, and yellow in the wine. Mean ± *SD* are reported. Means in a row with different superscripts (^a, b^) are significantly different at *p* < .05.

Flavonoids were present in fruit wine with *L. javanica* extract. This could be attributed to the addition of *L. javanica* extract in the must. Madzimure et al. (2011)identified a range of flavonoids and phenolic glycosides in *L. javanica* which include crassifolioside 11, luteolin 12, diosmetin 13, chrysoeriol 14, tricin 15, isothymusin 16, eupatorin 17, 5‐dimethyl noboletin 18, genkwanin 19, salvigenin 20, and an alkaloid xanthine 22. Furthermore, flavonoids have been found to possess many pharmacological properties that include anticancer, antibacterial, antioxidant, anti‐inflammatory, and antiviral (Kumar & Pandey, 2013) which are important in human health. The flavonoids, apigenin 7 present of *L. javanica* has been reported to prevent HIV‐1 activation by inhibiting the virus from the process of transcription (Critchfield et al., 1996) and luteolin 12 has been found to fight against herpes simplex virus in the presence of another flavonoid kaempferol (Kumar & Pandey, 2013).

Also, the increase in total phenol in the wine could be attributed to the presence of nonphenolic compounds in the fermenting substrate, that is, the bound soluble conjugated or insoluble phenolic acids in the cell walls of raw materials which could be converted into phenolic compounds by the action of esterase enzyme produced by the yeast (Tchabo et al., 2017). This improvement in phenolic content in the produced wine with *L. javanica* is in agreement with other researched done on the fermentation of apple‐herb wine (Lee et al., 2013).

The color properties of the produced wine are shown in Table 3. Results on wine color indicate that shade/brightness of the wine is higher than color intensity and color density. The observed color in the wine might have been produced from the reactions of anthocyanins and phenolic compounds present in the *L. javanica* extract during fermentation as well as during the wine aging (Tsegay and Gebremedhin (2019)). The yellow color of the *U. kirkiana* fruit juice and the added light brown color of *L. javanica* extract could have produced the yellowish color of the wine during the fermentation process (Table 3).

Fruit juice substrates with low sugar content produce wine with lower alcohol content after fermentation. In the study, the sugar content was adjusted by adding sucrose to the must. The produced wine had alcohol (ABV %) of 10.2 (Table 3). This could be explained by the production of ethanol during the fermentation process. In wine production, ethanol is the main alcohol produced and it normally determines the alcohol content. The alcohol content was higher than wines fermented from apple tea substrate (7.9 to 8.19% v/v) (Kumar et al., 2016). The differences in the composition of the substrate and method of the fermentation process used could have affected the alcohol content of the wines.

Sulfur dioxide is a common preservative still being used in wine production as an antioxidant and antimicrobial (Velić et al., 2018). Free sulfur dioxide content was 58.1 ± 1.2 and 50.2 ± 2.1 mg/L in wine with added *L. javanica* extract and control, respectively. The free SO_2_ in the prepared wine was higher than the wine produced from orange fruit juice (8.2 mg/L) (Kelebek et al., 2009) but it was in the acceptable level. The International Organization of Vine and Wine, formerly the Organisation Internationale de la Vigne et du Vin, has set an acceptable level of up to 150 mg/L for free SO_2_ content in wine (OIV, 2017). The free SO_2_ was introduced as potassium thiosulfate into the must. The potassium thiosulfate was added to inhibit the growth of some wild yeast species and bacteria related to wine spoilage. Sulfur dioxide is also important in inhibiting the activity of polyphenol oxidase during wine production (Guerrero & Cantos‐Villar, 2015). As a result, oxidative and unwanted spontaneous fermentation processes are both controlled by the action of sulfur dioxide (Ribereau‐Gayon et al., 2006). Additionally, sulfur dioxide reduces the rate of phenolic polymerization and loss of color during wine aging (Santos et al., 2012). However, consumption of SO_2_ in wines can have adverse health effects such as abdominal pain and diarrhea (Ferrer‐Gallego et al., 2017). It was therefore important in this study to regulate the addition of potassium thiosulfate (70 mg/L), a source of SO_2_ in the wine.

The antioxidant activity was 72.1 ± 0.1 and 46.3 ± 0.2 in wine with added *L. javanica* extract and control, respectively. This could be attributed to the presence of dietary phytonutrients such as phenolics and vitamins. Shikanga et al. (2010) reported an antioxidant activity with an EC_50_ value of 358 μg/mL for *L. javanica* leaf infusions and a total phenolic content of 14.8 mg GEA/ml of dry weight. More so, the observed high antioxidant activity in the *L. javanica* leaf extract and subsequently in the produced wine could be attributed to the high levels of verbascoside 2 (1.5 mg/g dry weight) found in the leaves of *L. javanica* (Olivier et al., 2010).

### 3.4 Sensory evaluation

Demographic characteristics have shown that men were the majority of participants that participated in the sensory evaluation (Tables 4). The majority of the sensory evaluation participants were aged 30–39 (*n* = 13).

**TABLE 4:** Characteristics of sensory participants

Characteristic	*n* (%)^a^
Gender	
Men	21 (70)
Women	9 (30)
Age‐groups (years)	
20–29	9 (30)
30–39	13 (43.3)
40–49	5 (16.6)
50+	3 (10)

^a^ Percentage of sample calculated using the total sample (*n* = 30).

Table 5 indicates number and percentages of panelists who gave the different ratings for the sensory attributes of the prepared wine with added *L. javanica* and control wine samples. Most of the panelists rated flavor and color of the produced wine with added *L. javanica* extracts as “good” compared control wine. The taste of the wine with added *L. javanica* extracts was rated as “average.” The control wine was rated “average” for flavor and color.

**TABLE 5:** Number and percentages of panelists who gave the different ratings for the sensory attributes evaluated (*n* = 30)

Food product	Rating	Taste	Flavor	Color	Overall acceptance
Wine with *Lippia* *javanica* extracts	Very bad	0^a^ (0.0)^b^	0 (0.0)	0 (0.0)	0 (0.0)
	Bad	5 (16.6)	4 (13.3)	4 (13.3)	2 (6.6)
	Average	13 (43.3)	8 (26.6)	9(30)	8 (26.6)
	Good	9 (30)	14(46.6)	12(40)	16(53.3)
	Very good	3 (10)	4(13.3)	5 (16.6)	4 (13.3)
Control	Very bad	0 (0.0)	1 (3.3)	2 (6.6)	0 (0.0)
	Bad	3 (10)	3(10)	1(3.3)	3 (10)
	Average	7(23.3)	16(53.3)	14(46.6)	13(33.3)
	Good	15(50)	6 (20)	10(33.3)	9(30)
	Very good	5 (16.6)	4 (13.3)	3 (10)	5 (16.6)

^a^ Number of subjects.

^b^ Percentage of the total number of participants. Acceptability rating 1–5:1 = very bad and 5 = very good.

The mean scores for the sensory attributes of the produced wine with added *L. javanica* extracts and control wine samples are presented in Table 6. The ratings of color in the produced wine could be attributed to a high concentration of phytochemicals from the added *L*. *javanica* extract. More so, phenolic acids, anthocyanins, flavonols, catechins, and other flavonoids could have been present in the fermenting substrate with *L. javanica* and impacted on the astringency and color of the wine (Kalkan Yildirim, 2006). The taste of the wine might have been influenced by the low residual sugar content, titratable acidity, and pH of the produced wine. This was supported by Tsegay and Gebremedhin (2019) who reported the influence of acidity on the taste of the wine produced from cactus pear with *Lantana camara* fruits. The flavor was liked by the panelists and this could be due to the presence of esters in wine. Velić et al. (2018) identified volatile compounds (esters, higher alcohols, organic acids) that could have contributed to the flavor of the wine. Zhu et al. (2016) reported the importance of minor volatile and nonvolatile compounds such as aldehydes, lactones, ketones, terpenes, and phenols on the flavor of wines. Furthermore, Tsegay and Gebremedhin (2019) indicated that ethanol produced during the fermentation of wine affects flavor and the overall quality of the wine.

**TABLE 6:** Mean scores for the sensory evaluation of wine with *Lippia* *javanica* and control wine samples (*n* = 30)

Food product	Taste	Color	Flavor	Overall acceptability
Wine with *L. javanica* extracts	3.9 ± 0.1	4.4 ± 0.1	4.5 ± 0.1	4.5 ± 0.1
Control	4.1 ± 0.1	3.9 ± 0.1	3.7 ± 0.2	3.9 ± 0.1
*p*‐value^a^	ns	<.05	<.05	<.05

Note

Mean ± *SD* are reported.

^a^ Mann–Whitney *U* test, ns = not significant.

## 4 CONCLUSION

The study showed that *L. javanica* leaves are good sources of phenols 14.3 ± 0.2 mg GAE/g, iron (77.3 ± 0.86 mg/100 g), potassium (1631 ± 0.05 mg/100 g), calcium (485.2 ± 0.1 mg/100 g), and carbohydrates. The produced wine had a pH, total sugar, TSS, total acidity, and vitamin C content of 3.1 ± 0.2, 3.0 ± 0.1 g/L, 4.8 ± 0.1%, 5.9 ± 0.01 g/L, and 4.4 ± 0.1 mg/100 g, respectively. Furthermore, the chemical and phenolic composition in the wine was significantly different (*p* < .05). More so, the alcohol (10.2 ± 0.1 ABV %,), free sulfur dioxide (58.1 ± 1.2 mg/L), antioxidant activity (73.1 ± 0.1 EC_50_ mg/L), and color intensity (40.4 ± 2.1 yellowish) were significantly different (*p* < .05) from control wine. This study noted the importance of the addition of *L. javanica* extracts as it complements vitamin C and flavonoids in the produced wine. The addition of *L. javanica* extract was able to improve the sensorial attributes of the wine. Also, it was noted that the wine had acceptable levels of sulfur dioxide that do not cause adverse health effects when consumed. Future research must evaluate the volatile acids, organic acids, and cytotoxicity analysis of the *L. javanica* extracts and the prepared wine.

## CONFLICTS OF INTEREST

The authors declare there is no conflict of interest.

## AUTHOR CONTRIBUTION

**Armistice Chawafambira:** Conceptualization (lead); Data curation (lead); Formal analysis (lead); Investigation (lead); Methodology (lead); Writing‐original draft (lead); Writing‐review & editing (lead).

## ETHICAL STATEMENT

This study does not involve any human or animal testing.

## Data Availability

The data of this research are available and will be provided on request by the authors.
